# Association between seasonal influenza vaccination and antimicrobial use in Japan from the 2015–16 to 2020–21 seasons: from the VENUS study

**DOI:** 10.1093/jac/dkad340

**Published:** 2023-10-28

**Authors:** Shinya Tsuzuki, Fumiko Murata, Megumi Maeda, Yusuke Asai, Ryuji Koizumi, Norio Ohmagari, Haruhisa Fukuda

**Affiliations:** AMR Clinical Reference Center, National Center for Global Health and Medicine, Tokyo, Japan; Disease Control and Prevention Center, National Center for Global Health and Medicine, Tokyo, Japan; Faculty of Medicine and Health Sciences, University of Antwerp, Antwerp, Belgium; Department of Health Care Administration and Management, Graduate School of Medical Sciences, Kyushu University, Fukuoka, Japan; Department of Health Care Administration and Management, Graduate School of Medical Sciences, Kyushu University, Fukuoka, Japan; AMR Clinical Reference Center, National Center for Global Health and Medicine, Tokyo, Japan; Disease Control and Prevention Center, National Center for Global Health and Medicine, Tokyo, Japan; AMR Clinical Reference Center, National Center for Global Health and Medicine, Tokyo, Japan; AMR Clinical Reference Center, National Center for Global Health and Medicine, Tokyo, Japan; Disease Control and Prevention Center, National Center for Global Health and Medicine, Tokyo, Japan; Department of Health Care Administration and Management, Graduate School of Medical Sciences, Kyushu University, Fukuoka, Japan

## Abstract

**Background:**

Seasonal influenza vaccination might be considered an antimicrobial resistance (AMR) countermeasure because it can reduce unnecessary antimicrobial use for acute respiratory infection by mitigating the burden of such diseases.

**Objectives:**

To examine the association between seasonal influenza vaccination and antimicrobial use (AMU) in Japan at the community level and to examine the impact of influenza vaccination on the frequency of unnecessary antimicrobial prescription for upper respiratory infection.

**Methods:**

For patients who visited any healthcare facility in one of the 23 wards of Tokyo, Japan, due to upper respiratory infection and who were aged 65 years or older, we extracted data from the Vaccine Effectiveness, Networking, and Universal Safety (VENUS) study database, which includes all claims data and vaccination records from the 2015–16 to 2020–21 seasons. We used the average treatment effect (ATE) with 1:1 propensity score matching to examine the association of vaccination status with frequency of antibiotic prescription, frequency of healthcare facility consultation, risk of admission and risk of death in the follow-up period of the same season (from 1 January to 31 March).

**Results:**

In total, 244 642 people were enrolled. Matched data included 101 734 people in each of the unvaccinated and vaccinated groups. The ATE of vaccination was −0.004 (95% CI −0.006 to −0.002) for the frequency of antibiotic prescription, −0.005 (−0.007 to −0.004) for the frequency of healthcare facility consultation, −0.001 (−0.002 to −0.001) for the risk of admission and 0.00 (0.00 to 0.00) for the risk of death.

**Conclusions:**

Our results suggest that seasonal influenza vaccination is associated with lower frequencies of unnecessary antibiotic prescription and of healthcare facility consultation.

## Introduction

Antimicrobial resistance (AMR) is a major global health issue.^1–4^ As part of the fight against AMR, the Global Action Plan on Antimicrobial Resistance, published by the WHO in 2015, strives to improve the awareness and understanding of AMR and strengthen knowledge through surveillance and research.^1^ Nonetheless, even with continuous efforts to combat AMR, it is still a substantial cause of disease burden in modern society.^4,5^ In contrast to expectations, previous studies have reported increases in the proportion of resistant organisms despite recent decreases in antimicrobial use (AMU).^6,7^ This suggests that alternative and multisectoral strategies should be explored and implemented to more effectively tackle AMR.

Vaccination can play a complementary role in AMR countermeasures.^8–10^ For instance, pneumococcal vaccination can itself reduce the incidence of penicillin-resistant *Streptococcus pneumoniae* (PRSP).^11,12^ Furthermore, a fall in carriers of drug-resistant bacteria may lead to their reduced circulation among the general population, which we would expect to have an additional impact on AMR.^10^ In contrast, seasonal influenza vaccination would seem not to be directly associated with antimicrobial-resistant bacteria because seasonal influenza is a viral infection. However, it may constitute an AMR countermeasure for two main reasons. One is a possible reduction in the collateral use of antibiotics. There is no doubt that vaccination helps to reduce the incidence of seasonal influenza.^13,14^ As a result, the number of complicated cases (e.g. influenza with bacterial pneumonia) will also fall, and this will decrease AMU.

The second reason is less frequent healthcare facility utilization due to vaccination. Because vaccination against seasonal influenza can reduce healthcare facility visits due to influenza-like illnesses among the general population, healthcare-seeking behaviour will change at the population level. The number of medically attended influenza cases will decrease under high coverage of the seasonal influenza vaccine, which can reduce the rate of inappropriate antimicrobial prescription.^15–17^ However, empirical evidence concerning the impact of seasonal influenza vaccination on inappropriate AMU thus far appears to be insufficient.

Against this background, the main objective of this study was to assess the impact of the seasonal influenza vaccine on the frequency of inappropriate antimicrobial prescription for upper respiratory infections (URIs) in the outpatient setting.

## Methods

### Data sources

For patients who visited any healthcare facility in one of the 23 wards in Tokyo, Japan, due to a URI and who were 65 years or older (because the Japanese government recommends seasonal influenza vaccination for individuals ≥65 years), we extracted data from the Vaccine Effectiveness, Networking, and Universal Safety (VENUS) study database.^18,19^ The data sources used to develop this system were: (i) the Basic Resident Register; (ii) the Vaccination Record System (VRS); and (iii) healthcare claims data.

We used Basic Resident Register to identify the base population. In Japan, the Basic Resident Register is the national registry of citizens and long-term residents, and includes each person’s resident registration number, name in kanji (Japanese logographic characters), name in kana (Japanese syllabic characters), birth date and sex. Resident registration numbers are unique identifiers for individual residents but are not commonly used in other databases.

The Japanese government developed the Basic Resident Register the VRS in 2021 to record the vaccination statuses of residents, including vaccination dates, locations, and types of vaccines. The VRS is a cloud-based system that municipal governments use to manage their vaccination-related data. The VRS includes resident registration numbers, which allow for direct data linkage with the Basic Resident Register.

Under Japan’s universal health insurance system, insurance is provided to all residents through three schemes: an employer-based insurance (for salaried employees of businesses), the National Health Insurance System, and the Latter-Stage Older Persons Health Care System. The region-based National Health Insurance System, in which municipal governments fulfil the role of insurer, provides coverage to the self-employed, unemployed, retired persons aged 65–74 years, and their dependents. The Latter-Stage Older Persons Health Care System, in which prefectural governments fulfil the role of insurer, provides coverage to all persons aged ≥75 years. For the VENUS study, healthcare claims data are collected from enrollees of the National Health Insurance System and the Latter-Stage Older Persons Health Care System by the participating municipalities. Each individual is assigned a unique research identification code based on their name, birth date and sex to allow their linkage across the data sources.

We set the study period as between 1 April 2015 and 31 March 2021. We extracted the information of people who were aged 65 years and older and had at least one record of healthcare claims data due to URI per year. We defined the baseline period as between 1 April and 31 December, and the follow-up period as between 1 January and 31 March, because seasonal influenza vaccination begins in October and ends in December in Japan.

### Data linkage

Ideally, the resident registration numbers in the Basic Resident Register would form the basis for linking the various data types as this would allow for the easy and accurate identification of all residents within a municipality. However, the lack of resident registration numbers in the healthcare claims data precludes the direct linkage of data. Therefore, we matched individuals in the three types of data through the matching of four criteria: name in kanji; name in kana; birth date; and sex. Each individual who could be linked across the data sources was assigned a unique research identification code to facilitate subsequent analyses. If there was more than one matching candidate for this matching algorithm, we excluded all such candidates from the final dataset.

### Statistical analysis

Descriptive statistics were used to describe the demographic characteristics of the patients in the database. Continuous variables are presented as median and IQR and categorical variables as the absolute number and proportion.

Next, we used modified Poisson regression analysis^20^ to examine the association of vaccination status with frequency of antibiotic prescription, frequency of healthcare facility consultation, risk of admission and risk of death in the follow-up period (from 1 January to 31 March) in the same season (from 1 April to 31 March). We excluded those who died during the baseline period.

We included people who had medical records of healthcare facility visits with a diagnosis of URI because we had no data on the background medical history of people without a record of healthcare facility visits, which meant that we could not calculate their Charlson comorbidity index (CCI). The diagnosis of URI was defined according to the International Classification of Diseases-10, as detailed in Table S1 (available as Supplementary data at *JAC* Online). Antimicrobial drugs were identified using the three-digit Therapeutic Category numbers defined by the Japanese Ministry of Health, Labour and Welfare between 611 and 625, which correspond to the A02, A07, J01, J02, J04, R01 and S01 codes of the Anatomical Therapeutic Chemical classification system established by the WHO.^21^ We divided the enrolled patients into two groups: unvaccinated and vaccinated. Both groups had at least one URI diagnosis documented in their medical records. We then conducted Poisson regression analyses to examine the association between each outcome variable and other factors. We included sex, age, CCI, frequency of consultation in the baseline period (from 1 April to 31 December), medical cost in the baseline period, vaccination status and year in a ‘frequency of antibiotic prescription’ model. Similarly, we included sex, age, CCI, frequency of consultation in the baseline period, medical cost in the baseline period, vaccination status and year in a ‘frequency of healthcare facility consultation’ model and sex, age, CCI, frequency of consultation in the baseline period, frequency of antibiotic prescription in the baseline period, medical cost in the baseline period, vaccination status and year in a ‘risk of admission’ model and ‘risk of death’ model.

Finally, we conducted 1:1 propensity score matching using data from the entire cohort. We assessed the impact of seasonal influenza vaccination on three outcomes—frequency of antibiotic prescription, risk of admission and risk of death in the same season—using the average treatment effect (ATE) with 1:1 propensity score matching.^22^ We adopted the nearest-neighbour matching method with the calliper set to 0.2. We adjusted for sex, age and number of healthcare facility visits in the baseline period, medical cost in the baseline period, comorbidities used to calculate CCI (myocardial infarction, congestive heart failure, peripheral vascular disease, cerebrovascular disease, dementia, chronic pulmonary disease, connective tissue disease, peptic ulcer disease, mild/severe liver disease, diabetes with/without chronic complication, hemiplegia, renal disease, any malignancy, metastatic solid tumour and HIV/AIDS) between the two unvaccinated and vaccinated groups. Replacement was not allowed. A standardized mean difference (SMD) greater than 0.1 was interpreted as a meaningful imbalance.^23^

Two-sided *P* values of <0.05 were considered statistically significant in all analyses. All statistical analyses were performed using R version 4.2.0.^24^

### Ethics

The study was approved by the Kyushu University Institutional Review Board for Clinical Research (approval no. 22114-01).

## Results

In total, 244 642 people were enrolled. For the data included in the modified Poisson regression model (i.e. the data before propensity score matching), 127 216 unvaccinated people and 117 426 vaccinated people were included. The vaccinated group were older than the unvaccinated group (median 82.0 versus 77.0 years) and a higher proportion were female (65.3% versus 61.2%). The vaccinated group also had a higher frequency of medical facility consultations in both the baseline and follow-up periods, a higher CCI and a lower proportion of admission. The details are shown in Table 1.

**Table 1.** Demographic characteristics of people with records of medically attended influenza-like illnesses (ILIs)

Data from 2015/16 season to 2020/21 season	Unvaccinated	Vaccinated	*P* value^a^
Number of people	127 216	117 426	
Year of vaccination, *n* (%)			NA
* *2015–16	22 299 (17.5)	20 721 (17.6)	
* *2016–17	22 431 (17.6)	21 318 (18.2)	
* *2017–18	24 263 (19.1)	21 295 (18.1)	
* *2018–19	23 124 (18.2)	21 709 (18.5)	
* *2019–20	20 567 (16.2)	21 636 (18.4)	
* *2020–21	14 532 (11.4)	10 747 (9.2)	
Age (years), median (IQR)	77.0 (72.0–82.0)	82.0 (77.0–87.0)	<0.001
Male, *n* (%)	49 338 (38.8)	40 758 (34.7)	<0.001
CCI, median (IQR)	0.0 (0.0–1.0)	0.0 (0.0–1.0)	<0.001
Frequency of consultation in baseline period, median (IQR)	0.0 (0.0–1.0)	1.0 (0.0–1.0)	0.017
Frequency of consultation in follow-up period, median (IQR)	1.0 (0.0–1.0)	1.0 (0.0–1.0)	0.073
Frequency of prescription in baseline period, median (IQR)	0.0 (0.0–0.0)	0.0 (0.0–0.0)	0.507
Frequency of prescription in follow-up period, median (IQR)	0.0 (0.0–0.0)	0.0 (0.0–0.0)	<0.001
Medical cost in JPY in baseline period, median (IQR)	0 (0–12 015)	0 (0–13 710)	0.068
Medical cost in JPY in follow-up period, median (IQR)	5050 (0–15 430)	5650 (0–17 910)	0.016
Admission in baseline period, *n* (%)	971 (0.8)	564 (0.5)	<0.001
Admission in follow-up period, *n* (%)	812 (0.6)	473 (0.4)	<0.001
Death in follow-up period, *n* (%)	43 (0.0)	27 (0.0)	0.121

JPY, Japanese Yen; NA, not available.

^a^Results of chi-squared test for categorical variables and Mann–Whitney *U*-test for continuous variables.

**Figure 1.**
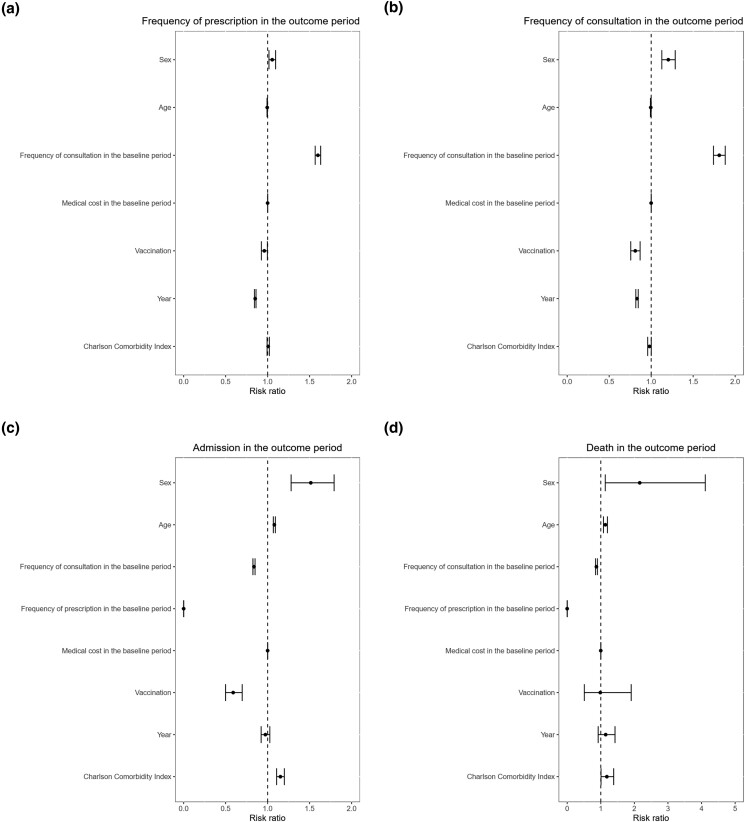
Results of modified Poisson regression analyses. (a) RR of each variable to frequency of antibiotic prescription. (b) RR of each variable to frequency of healthcare facility consultation. (c) RR of each variable to risk of admission. (d) RR of each variable to risk of death.

Modified Poisson regression analyses showed that seasonal influenza vaccination was associated with a lower risk ratio (RR) for the frequency of antibiotic prescription (RR 0.98, 95% CI 0.96–1.0, *P* = 0.022), higher RR for the frequency of healthcare facility visits (RR 1.12, 95% CI 1.11–1.12, *P* < 0.001), lower RR for admission (RR 0.51, 95% CI 0.48–0.54, *P* < 0.001) and lower RR for death (RR 0.39, 95% CI 0.30–0.51, *P* < 0.001). The results of the modified Poisson regression analyses are shown in Figure 1.

**Figure 2.**
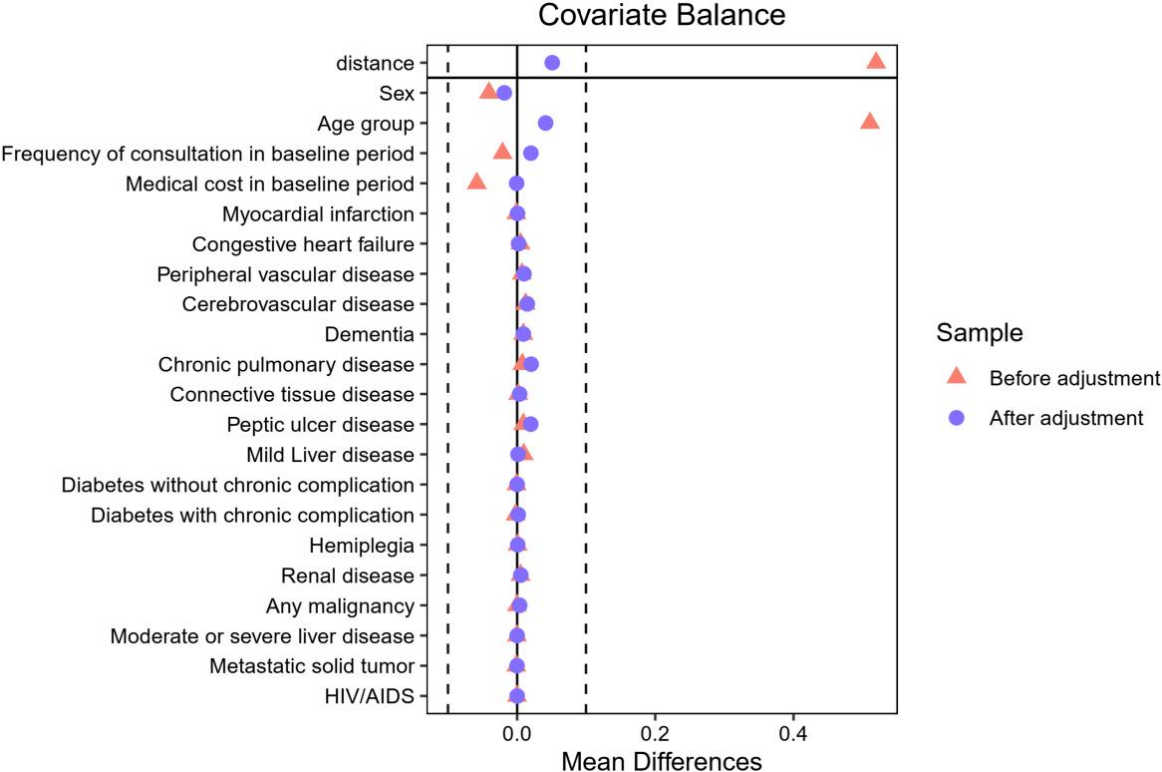
Balance of covariates before and after propensity score matching. Red squares represent data before adjustment and blue circles represent data after adjustment. Vertical dashed lines represent 0.10. This figure appears in colour in the online version of *JAC* and in black and white in the print version of *JAC*.

As for ATE estimation with propensity score matching, the matched data included 101 734 people in each of the unvaccinated and vaccinated groups (i.e. 203 468 of the 244 642 participants were included in the matched cohort). Chi-squared tests showed a higher frequency of healthcare facility consultation in both the baseline and follow-up periods in the vaccinated group but a higher number of admissions in both the baseline and follow-up periods in the unvaccinated group. The matched data are shown in Table 2 and the balance of matched data between the vaccinated and unvaccinated groups is shown in Figure 2.

**Table 2.** Demographic characteristics of the matched cohort

Data from 2015/16 season to 2020/21 season	Unvaccinated	Vaccinated	*P* value^a^	SMD
Number of patients	101 734	101 734		
Year of vaccination, *n* (%)			NA	
* *2015	18 909 (18.6)	20 397 (20.0)		
* *2016	18 308 (18.0)	20 773 (20.4)		
* *2017	20 016 (19.7)	20 666 (20.3)		
* *2018	18 197 (17.9)	18 353 (18.0)		
* *2019	14 815 (14.6)	14 938 (14.7)		
* *2020	11 489 (11.3)	6607 (6.5)		
Age (years), median (IQR)	82.0 (77.0–87.0)	82.0 (77.0–87.0)	<0.001	0.042
Male, *n* (%)	38 837 (38.2)	36 951 (36.3)	<0.001	0.038
CCI, median (IQR)	0.0 (0.0–0.0)	0.0 (0.0–0.0)	0.256	0.005
Frequency of consultation in baseline period, median (IQR)	0.0 (0.0–0.0)	0.0 (0.0–0.0)	0.002	0.020
Frequency of consultation in follow-up period, median (IQR)	0.0 (0.0–0.0)	0.0 (0.0–0.0)	<0.001	0.016
Frequency of prescription in baseline period, median (IQR)	0.0 (0.0–0.0)	0.0 (0.0–0.0)	<0.001	0.035
Frequency of prescription in follow-up period, median (IQR)	0.0 (0.0–0.0)	0.0 (0.0–0.0)	0.831	0.003
Medical cost in JPY in baseline period, median (IQR)	0 (0–0)	0 (0–0)	0.002	0.001
Medical cost in JPY in follow-up period, median (IQR)	0 (0–0)	0 (0–0)	<0.001	0.008
Admission in baseline period, *n* (%)	1048 (1.0)	739 (0.7)	<0.001	0.033
Admission in follow-up period, *n* (%)	290 (0.3)	191 (0.2)	<0.001	0.020
Death in follow-up period, *n* (%)	16 (0.0)	17 (0.0)	1.000	0.001

JPY, Japanese Yen; NA, not available.

^a^Results of chi-squared test for categorical variables and Mann–Whitney *U*-test for continuous variables.

The ATE of vaccination was −0.004 (95%CI −0.006 to −0.002) for the frequency of antibiotic prescription, −0.005 (−0.007 to −0.004) for the frequency of healthcare facility consultation, −0.001 (−0.002 to −0.001) for the risk of admission and 0.00 (0.00–0.00) for the risk of death.

## Discussion

This study demonstrated that seasonal influenza vaccination in the elderly population might reduce the frequency of antimicrobial prescription for URIs in the outpatient setting at the population level. To the best of our knowledge, little quantitative evidence has been published on the impact of seasonal influenza vaccination on AMU in Japan. Our results suggest that the seasonal influenza vaccine might have indirect benefit for not only preventing influenza-like illnesses, but also as a countermeasure against AMR.

As mentioned above, several previous studies reported this benefit as one of the countermeasures for AMR. For instance, Muller-Pebody *et al*.^17^ concluded that a live-attenuated influenza vaccine programme for pre-school age children had an inverse association with antibiotic prescription at the general physician level, while He *et al*.^25^ showed that influenza vaccine effectively reduced influenza-like illnesses and associated antibiotic prescriptions in healthy adults younger than 65 years old. According to Klein *et al*.,^26^ increased influenza vaccine uptake is associated with community-level reductions in AMU at the population level. Although Buckley *et al*.^27^ have claimed that the evidence is still weak and controversial, our results largely concur with those of previous studies and support the beneficial influence of the seasonal influenza vaccine on reducing unnecessary AMU.

On the other hand, the seasonal influenza vaccine showed a positive association with the frequency of healthcare facility consultation in the same season by modified Poisson regression analysis, which can be partially explained by the vaccination itself because people in Japan must visit a healthcare facility to be vaccinated. In addition, people who wish to be vaccinated may have higher health awareness than those who do not want to be vaccinated.^28–30^ This might strengthen our interpretation that seasonal influenza vaccination is beneficial for reducing inappropriate AMU because the people in the vaccinated group were prescribed fewer antibiotics despite more opportunities for antibiotics to be prescribed than those in the unvaccinated group.

However, we should take note that the ATE of vaccination on the frequency of healthcare facility consultation in the follow-up period was negative. This result suggests another interpretation, which is that vaccination reduces unnecessary healthcare facility visits and consequently reduces antimicrobial prescriptions. Both interpretations further support the benefits of vaccination because it acts in the direction of fewer antimicrobial prescriptions. This study also demonstrated that seasonal influenza vaccination might be associated with a lower RR for admission due to influenza-like illnesses. Needless to say, this is the original expected effect of the seasonal influenza vaccine and has already been reported repeatedly;^31–34^ accordingly, our findings reinforce the previous results. Although our matched cohort showed no statistically significant difference between the vaccinated and unvaccinated groups in relation to the risk of death, this is attributable to the small number of positive outcomes (i.e. the number of deaths in the follow-up period was small).

Our results suggested that the year of vaccination might also have had an impact on the main outcome (frequency of consultation in the follow-up period). The difference in the prevalent strain of influenza in each season and the variations in vaccine efficacy would likely impact the presentation of symptoms. In addition, the emergence of COVID-19 might also have had an impact on the outcome since the Japanese government recommended that the general population not go outside, which resulted in the less frequent use of healthcare facilities.^35^

Although our findings might be another reason to recommend seasonal influenza vaccination for the general population, we should interpret them with care, giving due consideration to their limitations. One of the major limitations of our results concerns the inclusion criteria. Because we included only an older population, we cannot know whether a similar effect would be seen if children or young adults were the target population for the vaccine. In addition, our data include claims data from only a single local authority in Tokyo, Japan, and therefore we should be careful when generalizing our results to other regions and/or countries.

Another limitation is a methodological issue related to adjustment for the background factors of each participant. As previous studies pointed out and we have already mentioned, the people in the vaccinated group may have higher health awareness and visit healthcare facilities with milder symptoms than unvaccinated people.^29,30,36^ If that is the case, the results of modified Poisson regression analyses and ATE estimation might be biased. However, we tried to adjust for this difference in health awareness and healthcare facility utilization by including the frequency of healthcare facility consultation and medical cost in the follow-up period in the modified regression model and propensity score calculation. Therefore, we believe that our results are sufficiently robust to warrant our discussion.

In conclusion, our results suggest that the seasonal influenza vaccine might help to reduce inappropriate AMU for URIs, although care should be taken when interpreting the results. This indirect benefit would be another reason to recommend seasonal influenza vaccination to the general population to enhance AMR countermeasures in society.

## Supplementary Material

dkad340_Supplementary_Data

## Data Availability

The data used in this study were acquired under agreements between Kyushu University and the participating municipalities, which stipulate that the data can be used only by authorized research institutions and cannot be shared with third parties.

## Supplementary data

Table S1 is available as Supplementary data at *JAC* Online.
