# Supplementary material for: Association between seasonal influenza vaccination and antimicrobial use in Japan from the 2015–16 to 2020–21 seasons: from the VENUS study
Source: J Antimicrob Chemother. 2023 Oct 28;78(12):2976–82. doi: 10.1093/jac/dkad340 (PMC10689917; doi:10.1093/jac/dkad340)
Supplement: dkad340_Supplementary_Data [file dkad340_supplementary_data.docx]

**Table S1. ICD-10 codes used to indicate the diagnosis of influenza-like illnesses in this study**

| ICD-10 codes | | | Diagnosis in Japanese Claims Code |
| --- | --- | --- | --- |
| J00 | Acute nasopharyngitis [common cold] | | |
|  | J00 | Acute nasopharyngitis [common cold] | Acute rhinitis (4609008) |
|  |  |  | Common cold (4609023) |
|  |  |  | Infective nasopharyngitis (8831632) |
|  |  |  | Infective rhinitis (4609014) |
|  |  |  | Nasopharyngitis (8832434) |
| J02 | Acute pharyngitis | | |
|  | J028 | Acute pharyngitis due to other specified organisms | Adenoviral pharyngitis (8845210) |
|  |  |  | Membranous pharyngitis (8840226) |
|  |  |  | Pseudomembranous pharyngitis (8832768) |
|  |  |  | Staphylococcal pharyngitis (8839824) |
|  |  |  | Viral pharyngitis (8830746) |
|  | J029 | Acute pharyngitis, unspecified | Acute pharyngitis (8832280) |
|  |  |  | Angina (8830401) |
|  |  |  | Catarrhal pharyngitis (8831360) |
|  |  |  | Infective pharyngitis (8831617) |
|  |  |  | Lower pharyngitis (4620006) |
|  |  |  | Pharyngitis (4629008) |
|  |  |  | Sore throat (7841001) |
|  |  |  | Ulcerative pharyngitis (8831058) |
|  |  |  | Upper pharyngitis (4620001) |
| J03 | Acute tonsillitis | | |
|  | J038 | Acute tonsillitis due to other specified organisms | Adenoviral tonsillitis (8845211) |
|  |  |  | Staphylococcal tonsillitis (8839832) |
|  |  |  | Viral tonsillitis (8830761) |
|  | J039 | Acute tonsillitis, unspecified | Acute adenoiditis pharyngitis (8832272) |
|  |  |  | Acute lacunar tonsillitis (4639020) |
|  |  |  | Acute tonsillitis (4639021) |
|  |  |  | Acute ulcerative tonsillitis (8832294) |
|  |  |  | Angina tonsillitis (8840010) |
|  |  |  | Habitual angina (8834526) |
|  |  |  | Habitual tonsillitis (8847346) |
|  |  |  | Lacunar angina (8836081) |
|  |  |  | Lingual tonsillitis (8836463) |
|  |  |  | Pseudomembranous tonsillitis (8832769) |
|  |  |  | Tonsillitis (4639033) |
| J04 | Acute laryngitis and tracheitis | | |
|  | J040 | Acute laryngitis | Acute edematous laryngitis (8832441) |
|  |  |  | Acute glottitis (4785003) |
|  |  |  | Acute laryngitis (8832342) |
|  |  |  | Acute subglottic laryngitis (4640009) |
|  |  |  | Acute ulcerative laryngitis (8832293) |
|  |  |  | Laryngitis (4640013) |
|  |  |  | Perilaryngitis (4640018) |
|  |  |  | Pseudomembranous laryngitis (4640021) |
|  | J041 | Acute tracheitis | Acute catarrhal tracheitis (8832299) |
|  |  |  | Acute tracheitis (8832320) |
|  |  |  | Hemorrhagic tracheitis (8834638) |
|  |  |  | Viral tracheitis (8830749) |
|  | J042 | Acute laryngotracheitis | Acute laryngotracheitis (8832344) |
| J05 | Acute obstructive laryngitis [croup] and epiglottitis | | |
|  | J050 | Acute obstructive laryngitis [croup] | Acute obstructive laryngitis (8832446) |
|  |  |  | Acute spasmodic laryngotracheitis (8832334) |
| J06 | Acute upper respiratory infections of multiple and unspecified sites | | |
|  | J060 | Acute laryngopharyngitis | Acute laryngopharyngitis (8832281) |
|  |  |  | Laryngopharyngitis (4650002) |
|  | J068 | Other acute upper respiratory infections of multiple sites | Acute pharyngotonsillitis (8832282) |
|  |  |  | Acute palatine tonsillitis (8832339) |
|  |  |  | Pharyngotonsillitis (8830672) |
|  |  |  | Pharyngotracheitis (8830654) |
|  | J069 | Acute upper respiratory infection, unspecified | Acute upper respiratory infection (4659007) |
| J20 | Acute bronchitis | | |
|  | J203 | Acute bronchitis due to coxsackievirus | Acute bronchitis due to coxsackievirus (8833756) |
|  | J204 | Acute bronchitis due to parainfluenza virus | Acute bronchitis due to parainfluenza virus (8839156) |
|  | J205 | Acute bronchitis due to respiratory syncytial virus | Acute bronchitis due to respiratory syncytial virus (8830134) |
|  | J206 | Acute bronchitis due to rhinovirus | Acute bronchitis due to rhinovirus (8840871) |
|  | J207 | Acute bronchitis due to echovirus | Acute bronchitis due to echovirus (8830860) |
|  | J208 | Acute bronchitis due to other specified organisms | Acute bronchitis due to human metapneumovirus bronchitis (8847902) |
|  |  |  | Viral bronchitis (4660001) |
|  | J209 | Acute bronchitis, unspecified | Acute bronchitis (4660009) |
|  |  |  | Acute laryngitis tracheobronchitis (8832345) |
|  |  |  | Acute recurrent bronchitis (8832429) |
|  |  |  | Acute tracheobronchitis (8832321) |
|  |  |  | Croupous bronchitis (8832840) |
|  |  |  | Exudative bronchitis (8834992) |
|  |  |  | Pseudomembranous bronchitis (4660012) |
|  |  |  | Subacute bronchitis (8830177) |
| J21 | Acute bronchiolitis | | |
|  | J210 | Acute bronchiolitis due to respiratory syncytial virus | Acute bronchiolitis due to respiratory syncytial virus (8830135) |
|  | J218 | Acute bronchiolitis due to other specified organisms | Acute bronchiolitis due to human metapneumovirus (8847903) |
|  | J219 | Acute bronchiolitis, unspecified | Acute bronchiolitis (8832358) |
| J22 | Unspecified acute lower respiratory infection | | |
|  | J22 | Unspecified acute lower respiratory infection | Acute (lower) respiratory (tract) infection (8832349) |
